# Causality of Chronic Suppurative Otitis Media: An Observational Study

**DOI:** 10.7759/cureus.9832

**Published:** 2020-08-18

**Authors:** Zafar Ayaz, Bakht Taj, Maher Sohail Yaseen, Uzma Ishaq, Talha Laique, Jahanzeb Malik, Adnan Baig, Komal Sakhawat

**Affiliations:** 1 Otolaryngology, Timergara DHQ Hospital, Timergara, PAK; 2 Otolaryngology, DHQ Hospital Daggar Buner, Buner, PAK; 3 Physiology, Dera Ghazi Khan Medical College, Dera Ghazi Khan, PAK; 4 Hematology and Medical Oncology, Fauji Foundation Hospital, Rawalpindi, PAK; 5 Pharmacology, Lahore Medical and Dental College, Lahore, PAK; 6 Cardiology, Rawalpindi Institute of Cardiology, Rawalpindi, PAK

**Keywords:** chronic otorrhoea, pseudomonas aeuginosa, klebsiella pneumoniae, otitis media, chronic suppurative otitis media, ear infection

## Abstract

Objective

Chronic otorrhoea is a disease of the ear that lasts for more than 6-12 weeks, through a perforated tympanic membrane. We sought to determine the cause of chronic suppurative otitis media (CSOM) at our institute.

Methodology

Ear swabs taken from enrolled patients (n=139) were sent for culture and sensitivity. Statistical analysis was done using Statistical Package for Social Sciences (SPSS), version 26 (IBM Corp., Armonk, NY)

Results

Common pathogenic bacteria in chronic otitis media observed were Pseudomonas aeruginosa 81 (58%) while Klebsiella pneumoniae was found in 58 (42%) of cases.

Conclusion

We concluded that Pseudomonas aeruginosa was the most common causative agent of CSOM at our institute.

## Introduction

The most common disease in ENT is chronic suppurative otitis media (CSOM). It has been a common health issue among children for centuries. Chronic otorrhoea is a disease of the ear that lasts for more than 6-12 weeks, through a perforated tympanic membrane. Globally, it affects almost 65-100 million people annually. About 60% of its sufferers experience significant hearing loss mainly because of it [1]. 

Its prevalence is not known in our region, although the overall prevalence rate of CSOM was found to be 6% [2]. In Saudi children, chronic ear infection is present in 1.5% of all ear infections [3]. It is equally distributed among males and females globally [4]. It is associated with complications like hearing impairment, disability, poor scholastic performance, intracranial infections, and acute mastoiditis. It is more common among developing countries. The most common complication of CSOM is acute mastoiditis [5].

Since the majority of these patients are seen by general practitioners, only a limited number of patients have their ear swabs sent for culture and sensitivity. Mostly the treatment is empirical [6]. 

Staphylococcus epidermidis, Corynebacterium spp., Streptococcus viridans, and Staphylococcus aureus are usually present as commensal flora on the skin of the external auditory canal [7,8]. Pseudomonas and Proteus spp. invade secondarily in cases of otitis externa due to breakage in the skin's natural defense mechanism. With an intact tympanic membrane, the culture swabs from middle ear mucosa show no bacterial growth.

When a defect in the tympanic membrane is present, the flora of the external auditory meatus can be isolated from the middle ear. In CSOM, bacteria may be gram-positive or gram-negative [7]. Complications related to CSOM are due to missed diagnosis and inadequate treatment. The complications include bloody discharge, facial palsy, vertigo, fever, irritability, headache, and deafness [9-11].

An effective treatment strategy for reducing the development of its complications is the need of the hour as well as to decrease the number of CSOM in ENT at our tertiary hospitals. In our country, due to the lack of registered cases of CSOM as well as its causative agents, there is a paucity of local studies. In light of this increasing burden, we conducted this study to determine the frequently encountered pathogens for CSOM.

## Materials and methods

A sample size of 139 patients with a 10% proportion of the common causative organisms using a 95% confidence interval and a 5% margin of error was calculated using a software. Data collection was started after ethical board approval and written consent was taken from all the participants. It was a descriptive cross-sectional study. Swabs were taken from the deep part of the meatus near to the tympanic membrane. They were sent for culture and sensitivity. All the results were recorded.

All the data were processed using the Statistical Package for Social Sciences (SPSS), version 26 (IBM Corp., Armonk, NY). Mean and standard deviation was calculated for age. Frequency and percentages were calculated for categorical variables. Bacterial pathogens were stratified among age and gender to see the effect modifiers.

## Results

A total of 139 patients were enrolled in the study. Among the patients, 92 (66.19%) were males and 110 (79%) were children of less than 10 years of age. Baseline characteristics and organisms are shown in Table 1.

**Table 1:** Baseline characteristics and organisms

Baseline Characteristics	
Age (years)	8.32 + 3.77
Males n(%)	92 (66.19%)
Children < 10 years n(%)	110 (79%)
Organisms	
Pseudomonas Aeruginosa n(%)	81(58%)
Klebsiella Pneumonae n(%)	58(42%)

Children less than 10 years of age were affected the most and Pseudomonas aeruginosa was the predominant organism seen in this age group (P-value = 0.01). Age-wise distribution of organisms is shown in Table 2.

**Table 2:** Age-wise distribution of the organisms

Age group	Pseudomonas Aeruginosa n(%)	Klebsiella Pneumonae n(%)
< 10 years	63(76.8%)	47(23.2%)
≥ 11 years	18(62%)	11(38%)

The males were infected more as compared to females and the organism seen the most on culture was Pseudomonas aeruginosa (P-value = 0.04). The gender distribution of organisms is shown in Table 3.

**Table 3:** Gender distribution of the organisms

Gender	Pseudomonas Aeruginosa n(%)	Klebsiella Pneumonae n(%)
Male	58(63%)	34(37%)
Female	24(51%)	23(49%)

## Discussion

CSOM is a chronic infection of the middle ear, with otorrhoea through a perforated tympanic membrane. It is the most common ear infection in children under the age of 10 years [12]. Our study confirms these findings. 

There is a variability in the number of participants recruited in the studies worldwide. Two studies enrolled only 68 patients for CSOM [13, 14]. Another study carried out in 1991 included 360 patients for CSOM [15]. We included 139 patients, keeping in mind the prevalence in our region. Males were 92 (66.19%) while females were 47 (33.81%) in our project. In contrast to this, females were more prone to contracting CSOM as demonstrated by one study [16]. 

In this study, patients were divided into two age groups i.e., less than equal to 10 years and more than or equal to 11 years. In contrast, one study carried in 2011 in Nepal included 6005 patients of CSOM but distribution varied with respect to age groups, ranging from less than 10 years to more than 50 years [17]. Among the 139 enrolled patients, the frequency of only two pathogens (Pseudomonas aeruginosa and Klebsiella pneumonae) was observed. In contrast, previous studies observed various aerobic and anaerobic pathogens for CSOM. Our findings were similar to one study which demonstrated Pseudomonas aeuruginosa as the predominant organism (50%) in CSOM [18].

This study has various limitations. It is a single-center study and there were only two organisms isolated in the swab cultures. No effect of antibiotics was observed in the study and no complications were seen. A large-scale multicenter study is needed in our region to accurately describe the causality of CSOM.

## Conclusions

This study helped in identifying the causality of the most common bacteria of CSOM at our setup. Almost all the patients had either of the two organisms, Pseudomonas aeruginosa or Klebsiella pneumonae. It will help in the appropriate prescription of antibiotics for the treatment of CSOM. To our knowledge, this is the first study to see the causal relationship of CSOM with the organism in our region.
